# Supplementary material for: Quantitative MRI reveals differences in striatal myelin in children with DLD
Source: eLife. 2022 Sep 27;11:e74242. doi: 10.7554/eLife.74242 (PMC9514847; doi:10.7554/eLife.74242)
Supplement: Transparent reporting form [file elife-74242-transrepform1.docx]

***eLife’s* transparent reporting form**

We encourage authors to provide detailed information *within their submission* to facilitate the interpretation and replication of experiments. Authors can upload supporting documentation to indicate the use of appropriate reporting guidelines for health-related research (see [EQUATOR Network](http://www.equator-network.org/%20)), life science research (see the [BioSharing Information Resource](https://biosharing.org/)), or the [ARRIVE guidelines](http://www.plosbiology.org/article/info:doi/10.1371/journal.pbio.1000412) for reporting work involving animal research. Where applicable, authors should refer to any relevant reporting standards documents in this form.

If you have any questions, please consult our Journal Policies and/or contact us: [editorial@elifesciences.org](mailto:editorial@elifesciences.org).

**Sample-size estimation**

- You should state whether an appropriate sample size was computed when the study was being designed
- You should state the statistical method of sample size computation and any required assumptions
- If no explicit power analysis was used, you should describe how you decided what sample (replicate) size (number) to use

Please outline where this information can be found within the submission (e.g., sections or figure legends), or explain why this information doesn’t apply to your submission:

This data was collected as part of a multimodal imaging study of language development, where we collected fMRI, diffusion, and structural scans. To maximise power across the different scans, our aim was to collect 80 datasets (and a minimum of 45) in each group (typically developing children, children with DLD) over the course of the study. This is also described in our registered report (Krishnan et al., 2021, NeuroImage).

In the process of grant submission, we calculated minimum sample sizes to give us 80% power to detect group differences in subcortical volumes on the basis of our pilot data (6 children with DLD and 12 controls). The minimum group sample was less than 20 per group for detecting differences in subcortical regions (including the caudate nucleus, nucleus accumbens, thalamus), with the exception of the putamen. However, this was a small pilot study, and the MPM method was novel. We therefore tried to maximise the numbers of children we could include in the analyses described in this study.

In the manuscript we clearly describe our data retention process (moving from the 175 children recruited into the BOLD study, (page 18), to the number of children who completed the MPM scans (N=148), page 18, to how we ended up our final sample size of 109 datasets after quality control (pg 20-21)).

**Replicates**

- You should report how often each experiment was performed
- You should include a definition of biological versus technical replication
- The data obtained should be provided and sufficient information should be provided to indicate the number of independent biological and/or technical replicates
- If you encountered any outliers, you should describe how these were handled
- Criteria for exclusion/inclusion of data should be clearly stated
- High-throughput sequence data should be uploaded before submission, with a private link for reviewers provided (these are available from both GEO and ArrayExpress)

Please outline where this information can be found within the submission (e.g., sections or figure legends), or explain why this information doesn’t apply to your submission:

This is a single experiment, and we did not have the resources to replicate these findings at this stage.

We have clearly described inclusion/ exclusion criteria in terms of participants and data quality in the manuscript (page 18 and page 20-21).

**Statistical reporting**

- Statistical analysis methods should be described and justified
- Raw data should be presented in figures whenever informative to do so (typically when N per group is less than 10)
- For each experiment, you should identify the statistical tests used, exact values of N, definitions of center, methods of multiple test correction, and dispersion and precision measures (e.g., mean, median, SD, SEM, confidence intervals; and, for the major substantive results, a measure of effect size (e.g., Pearson's r, Cohen's d)
- Report exact p-values wherever possible alongside the summary statistics and 95% confidence intervals. These should be reported for all key questions and not only when the p-value is less than 0.05.

Please outline where this information can be found within the submission (e.g., sections or figure legends), or explain why this information doesn’t apply to your submission:

We have fully described statistical analysis in our methods section, highlighting the use of the software packages across the brain (pages 18-21).

In most cases, the differences reported here are statistical maps across the brain, and we have used standard methods to report these differences , and used corrected thresholds (page 21). We have also made maps available on Neurovault, which allows readers to view unthresholded maps and change these settings.

We have also presented raw data in figures where possible (i.e. in Figures 1 & 4). We have reported exact p-values where useful, the only exception is in the summary statistics for descriptive data (Supplementary Table 1) where this would become excessive and not necessarily helpful, however, the raw data is available on the OSF for anyone who wants to reproduce this analyses.

(For large datasets, or papers with a very large number of statistical tests, you may upload a single table file with tests, Ns, etc., with reference to sections in the manuscript.)

**Group allocation**

- Indicate how samples were allocated into experimental groups (in the case of clinical studies, please specify allocation to treatment method); if randomization was used, please also state if restricted randomization was applied
- Indicate if masking was used during group allocation, data collection and/or data analysis

Please outline where this information can be found within the submission (e.g., sections or figure legends), or explain why this information doesn’t apply to your submission:

On page 18 we describe our criteria for allocating participants into the different groups (typically developing, DLD, and history of speech and language problems).

**Additional data files (“source data”)**

- We encourage you to upload relevant additional data files, such as numerical data that are represented as a graph in a figure, or as a summary table
- Where provided, these should be in the most useful format, and they can be uploaded as “Source data” files linked to a main figure or table
- Include model definition files including the full list of parameters used
- Include code used for data analysis (e.g., R, MatLab)
- Avoid stating that data files are “available upon request”

Please indicate the figures or tables for which source data files have been provided:

We have made statistical maps available on Neurovault (https://neurovault.org/collections/DUGBDBPH/).

We have also made the data from regions of interest in Figure 1 and Figure 4 available on our OSF repository in summary tables (https://osf.io/d93gq/?view_only=c48c989c574a49cba6eba1c413f185bb), in addition to other data that describes our sample.
